# A Lymphatic Perspective on Obesity and Inflammatory Arthritis: New Disease-Modifying Potential in Rheumatology

**DOI:** 10.3390/jcm14217641

**Published:** 2025-10-28

**Authors:** Hannah den Braanker, Eline S. van der Valk, Radjesh J. Bisoendial

**Affiliations:** 1 Department of Rheumatology and Clinical Immunology, Maasstad Hospital, 3079 DZ Rotterdam, The Netherlands; 2 Department of Endocrinology, Erasmus Medical Centre, 3015 GD Rotterdam, The Netherlands; 3 Department of Endocrinology, Utrecht University Medical Centre, 3584 CX Utrecht, The Netherlands; 4 Department of Developmental Biology, Erasmus Medical Centre, 3015 GD Rotterdam, The Netherlands

**Keywords:** inflammatory arthritis, obesity, lymphatics, rheumatoid arthritis, psoriatic arthritis, gout, cytokines

## Abstract

Recent studies show that obesity significantly increases disease severity and progression in several forms of inflammatory arthritis, including rheumatoid arthritis (RA), psoriatic arthritis (PsA) and gout. Obesity increases the risk for developing inflammatory arthritis. Similarly, obesity negatively impacts disease severity and treatment outcomes. The underlying mechanisms driving these relationships are not fully understood. One emerging area of investigation is the role of the lymphatic vasculature. Obesity profoundly impacts lymphatic function. Excess adipose tissue can compress and disrupt lymphatic vessels, leading to impaired flow and drainage. Additionally, obesity-associated inflammation and metabolic dysregulation have been linked to lymphatic endothelial cell dysfunction, further compromising transport and immunoregulatory capacities. This impairment fosters an environment for the accumulation of inflammatory cells and mediators, sustaining chronic inflammation. In this review, we will provide new perspectives on the detrimental triangle of obesity, lymphatic dysfunction, and inflammation in chronic inflammatory arthritis and find new starting points for therapeutics.

## 1. Introduction

The prevalence of obesity has reached pandemic proportions [1], and the prevalence of its complications likewise increases [2]. Obesity drives higher mortality through its devastating health consequences—including cardiovascular disease and increased cancer risk—but also fuels a growing epidemic of non-communicable diseases [3]. Among these, autoimmune diseases and inflammatory arthritis have emerged as particularly intriguing. Inflammatory arthritis encompasses a spectrum of diseases—including rheumatoid arthritis (RA), psoriatic arthritis (PsA), and gout—that collectively affect over 350 million individuals worldwide [4,5,6]. The prevalence of gout, the most common inflammatory arthritis, increased 20% in recent decades [4]. Similarly, the prevalence of RA, the most common autoimmune inflammatory arthritis, increased by 15% over the past decades [5]. These rising burdens parallel the global obesity epidemic, and obesity seems to be one of the largest contributors to the rising incidence of inflammatory arthritis [7]. Furthermore, patients with inflammatory arthritis who also have obesity experience higher disease activity scores and require more aggressive treatment regimens [7,8,9,10,11]. Understanding the mechanistic links between arthritis and obesity has therefore become critical for improving patient outcomes.

Current understanding of obesity’s impact on inflammatory arthritis centers on adipose tissue as an endocrine organ producing systemic pro-inflammatory mediators including TNF-α, IL-6, and leptin, which have the potential to fuel local synovial inflammation [12]. However, the observation that inflammatory arthritis develops in only a subset of individuals with obesity indicates that additional pathophysiological mechanisms must be operative. An underexplored yet potentially critical factor is the disruption of lymphatic function, a system responsible not only for lipid transport and distribution but also for the immune cell migration, and clearance of inflammatory mediators like immune complexes and cellular debris from tissues, including synovial spaces [13]. Obesity compromises lymphatic function through multiple mechanisms. Mechanical compression from expanding adipose tissue impedes lymphatic flow. Meanwhile, metabolites from visceral adipose tissue and pro-inflammatory cytokines directly affect multiple aspects of lymphatic function. These include damaging the endothelial glycocalyx, the carbohydrate-rich surface layer critical for mediating mechanotransduction of shear stress and maintaining lymphatic barrier function, impairing lymphatic valve integrity, and disrupting the immunoregulatory functions of lymphatic endothelial cells and lymph nodes [14,15,16,17]. Notably, correct formation of lymphatic valves and lymphatic vessels is governed by a complex interplay of genetic and epigenetic modifications, which might influence individual susceptibility to obesity-induced lymphatic dysfunction [18,19]. When obesity-induced lymphatic dysfunction coincides with inflammatory triggers, the resulting drainage failure may create a “perfect storm”. Thus, pathological immune cells and non-cellular inflammatory mediators accumulate in joints and periarticular tissues, overwhelming local resolution mechanisms and establishing self-perpetuating inflammatory circuits that become increasingly independent of initial triggers. Weight reduction can reverse those lymphatic changes in early stages in murine models [16,20]. While early-stage lymphatic dysfunction may be reversible through weight reduction, delayed intervention risks permanent lymphatic architectural damage, underscoring the critical importance of early therapeutic intervention [16,21]. The advent of incretin analogs, such as glucagon-like-peptide 1 agonists, represents a transformative opportunity for early intervention in inflammatory arthritis. However, it is critical to emphasize that these agents are not standalone solutions but rather components of a comprehensive, multidisciplinary therapeutic strategy. This review aims to synthesize current evidence on the role of the lymphatic–metabolic axis in inflammatory arthritis and explore potential therapeutic interventions. Optimal management of obesity-associated inflammatory arthritis requires coordinated implementation of lifestyle modifications (including structured dietary intervention and progressive physical activity programs) [22], conventional disease-modifying antirheumatic drugs (DMARDs) and/or biological therapies targeting specific inflammatory pathways, and emerging metabolic interventions such as incretin analogs. We propose that lymphatic dysfunction is a mechanism linking obesity to arthritis development and treatment failure. By integrating metabolic optimization through lifestyle and pharmacological interventions, clinicians may achieve synergistic benefits from weight reduction and metabolic improvement restoring lymphatic architecture and function, while immunomodulatory therapies address the inflammatory burden. By reframing obesity-related arthritis as partly a reversible lymphatic disorder with architectural and functional alterations that is amenable to metabolic intervention, this perspective challenges current treatment paradigms and offers hope for the growing population of patients failing conventional therapies.

## 2. The Role of Obesity in Developing Rheumatoid Arthritis

The impact of obesity on the emergence and clinical course of rheumatoid arthritis may differ based on the immunophenotype and phase, as well as obesity-related factors, like severity and distribution of adipose tissue (central/visceral versus peripheral/subcutaneous). Central obesity, characterized by excess visceral adipose tissue around abdominal organs, is considered more metabolically harmful than peripheral obesity due to visceral fat’s greater production of inflammatory cytokines [23,24]. A recent well-designed Mendelian randomization study by Fan et al. shows that genetically determined obesity significantly increases the risk of psoriasis (OR 1.52), myasthenia gravis (OR 1.41), inflammatory bowel disease, and seropositive rheumatoid arthritis (RA) (OR 1.23). Notably, the effect sizes were modest, and seronegative RA showed no significant association [25]. For context, RA is traditionally subdivided based on the presence or absence of autoantibodies: seropositive RA features anti-citrullinated protein antibodies (ACPA) and/or rheumatoid factor (RF), while seronegative RA lacks these markers. This distinction is crucial as long-term outcomes and underlying pathophysiological mechanisms differ [26]. This genetic evidence in seropositive RA is partially supported by a large prospective UK Biobank cohort study showing that obesity increases the risk of both seropositive and seronegative RA, with hazard ratios of 1.52 and 1.47, respectively. Abdominal obesity—measured by waist circumference rather than body mass index (BMI) alone—showed a hazard ratio of 1.58, where each 2.5 kg/m^2^ increase in BMI was associated with a 9% higher risk of RA [27]. However, these observational findings are potentially limited by residual confounding from shared environmental or behavioral factors. Indeed, a large Mendelian randomization study by Li et al., using multiple publicly available datasets, including the UK Biobank and FinnGen studies, surprisingly found that the association between BMI and RA risk was attenuated when accounting for smoking and drinking behaviors [28]. Earlier population-based studies from Rochester, Minnesota, suggested that obesity accounts for approximately 52% of the increased RA incidence between 1985 and 2007 [7]. The Nurses’ Health Study II, initiated in 1989, reported that weight gain of 2–10 kg already increased the relative risk for RA (RR 1.98), while weight gain exceeding 20 kg showed relative risks of 3.81 for both seropositive and seronegative RA [29]. Yet these dramatic attributions to RA risk require consideration of concurrent changes in diagnostic criteria and increased disease awareness.

Most studies on obesity’s effect on RA risk involved women. In a meta-analysis, Ohno et al. determined that the association between obesity and RA development is restricted to women [30]. Furthermore, Hedström et al. even found an inverse correlation between BMI and ACPA-positive RA in men [31], suggesting potential sex-specific immunological or hormonal interactions. Another intriguing factor is the timing of when obesity confers the greatest risk for RA development. In clinically suspect arthralgia (CSA)—patients identified in rheumatology as being at risk for RA development during an asymptomatic phase—obesity was not identified as a risk factor for progression to definite RA. However, patients with CSA already had increased BMI compared to the healthy population [32]. Interestingly, a recent Korean study found that BMI ≥ 25 kg/m^2^ with a normal waist circumference, was associated with a lower risk for RA, especially in women [33]. Thus, our understanding of the relationship between RA and obesity remains incomplete. Moreover, the divergent associations between obesity and seropositive versus seronegative RA suggest fundamentally different pathophysiological mechanisms underlying these disease subtypes.

## 3. The Role of Obesity in Developing Psoriatic Arthritis

In psoriatic arthritis (PsA)—a chronic inflammatory arthritis affecting joints and entheses (tendon-bone attachment sites) developing in 10–40% of psoriasis patients—the relationship with obesity presents intriguing but inconsistent findings [34]. Soltani-Arabshahi et al. demonstrated that early adulthood obesity (ages 18–20) strongly associates with PsA development (OR 3.12), suggesting a critical window of vulnerability [35]. However, this retrospective study relied on self-reported weight history, introducing potential recall bias. A larger prospective cohort by Eder et al. showed only a modest relative risk (RR 2.02) for BMI’s effect on PsA development, raising questions about the true magnitude of this association [36]. The Nurses’ Health Study II revealed a striking dose–response relationship: compared with BMI < 25.0 kg m^2^, the relative risk was 1.83 for BMI 25.0–29.9 kg m^2^, 3.12 for BMI 30.0–34.9 kg m^2^, and 6.46 for BMI ≥ 35.0 kg m^2^ [37,38]. Yet these studies predominantly included white, female healthcare professionals with a predominant standing profession, limiting generalizability. A UK electronic health records study confirmed that PsA incidence increased with BMI (BMI 25.0–29.9 kg m^2^ OR 1.79; BMI 30.0–34.9 kg m^2^ OR 2.10 and BMI > 35.0 kg m^2^ OR 2.68) among psoriasis patients, though distinguishing whether obesity mechanistically drives immunophenotypic transition or represents a shared risk factor with common upstream drivers remains challenging [39]. Critically, the underlying mechanisms governing psoriasis-to-PsA transition remain poorly characterized, making obesity’s role currently difficult to determine [34].

## 4. The Role of Obesity in Gout

The relationship between obesity and gout exemplifies bidirectional metabolic–inflammatory crosstalk in crystal-induced arthropathy. Gout, a crystal-induced arthropathy caused by monosodium urate (MSU) precipitation, has historically been termed the “disease of kings” due to its association with dietary excess and obesity [40]. The global burden of gout has risen dramatically, with age-standardized prevalence increasing by 22.5% between 1990 and 2020 to affect 55.8 million people worldwide, paralleling the obesity pandemic and projected to continue rising through 2050. In the case of gout, obesity contributes around 34% to this rising incidence [4]. Recent evidence reveals that the relationship extends beyond simple causality: obesity increases gout risk through multiple metabolic pathways, while gout-associated inflammation, pain-related immobility, and hyperuricemia treatment (particularly corticosteroids) can paradoxically promote further weight gain [41,42].

Adipose tissue dysfunction, particularly visceral fat accumulation, drives gout pathogenesis through mechanisms extending beyond mechanical weight effects. Lee et al. demonstrated that visceral fat obesity (VFO), defined as visceral fat area > 100 cm^2^, was present in 71.8% of gout patients compared to 41.2% of controls, with VFO remaining an independent risk factor (OR 2.49) even after adjusting for BMI [43]. Remarkably, among individuals with BMI < 25 kg/m^2^, 47.4% of gout patients exhibited visceral fat obesity compared to 27.3% of controls, despite comparable total fat mass. This indicates that visceral adiposity drives gout pathogenesis through mechanisms independent of overall weight, rather related to the chronic low-grade inflammation than to mass effects of obesity. A comprehensive NHANES analysis (2001–2018) of 29,310 participants confirmed that central obesity indices—body roundness index (BRI) and weight-adjusted waist index (WWI)—demonstrated superior diagnostic efficacy for hyperuricemia compared to BMI alone (AUC 0.669 for BRI vs. 0.664 for BMI), with each unit increase in WWI associated with a 72% increased risk of hyperuricemia [44].

The obesity–gout association exhibits marked heterogeneity across demographic and clinical subgroups, necessitating risk-stratified approaches. First, sex differences are pronounced. While gout predominantly affects men (male:female ratio ~3:1) [4], obesity confers greater relative risk in women, particularly postmenopausal women who lose the uricosuric effect of estrogen [42,45]. Early-life obesity (ages 18–30) was also associated with an earlier onset of gout, particularly in men [46]. Pacific Islander, Māori, and African American populations demonstrate both elevated baseline hyperuricemia and greater obesity-attributable gout risk compared to white populations, likely reflecting genetic variants in urate transporters (ABCG2, SLC2A9) interacting with metabolic factors [47]. Lastly, comorbidity burden critically modulates risk: metabolic syndrome components combined with obesity seem to produce multiplicative rather than additive effects [48].

## 5. Treatment Response in Inflammatory Arthritis

The negative impact of obesity on therapeutic outcomes in inflammatory arthritis is well-established, though the magnitude and mechanisms vary by disease phenotype and treatment modality. Personalized treatment strategies accounting for all these factors may be necessary to optimize outcomes in this high-risk population.

### 5.1. Rheumatoid Arthritis: Divergent Mechanisms in Seropositive Versus Seronegative Disease and Treatment Specific Interactions

In general, meta-analyses demonstrate that RA patients with obesity have 47–60% higher odds of failing to achieve remission compared to normal-weight patients, though these analyses often conflate different remission criteria and treatment modalities [9,10]. The heterogeneity in outcome measures—ranging from DAS28 remission to Boolean criteria—complicates interpretation and explains varying effect sizes across studies.

In ACPA-positive RA—generally considered the more “autoimmune” phenotype due to autoantibodies—obesity’s impact appears particularly pronounced, with patients exhibiting persistently higher disease activity scores (DAS28 difference of +0.43), elevated CRP levels, and increased swollen joint counts throughout their disease course compared to ACPA-negative patients [49]. This suggests that obesity amplifies the classic autoimmune cascade: adipose-derived mediators (leptin, IL-6, TNF) enhance B cell activation, autoantibody production, and immune complex-mediated inflammation, creating a treatment-resistant milieu [50,51,52]. The NORD-STAR study reinforced obesity as an independent predictor of poor treatment response, specifically in early RA, with obesity conferring a 2.4-fold increased risk of treatment failure [8,11]. In the NORD-STAR study around 80% was ACPA or rheumatoid factor positive RA. Recent outcomes research refines this picture on the cardiovascular axis: BMI was associated with higher cardiovascular risk among ACPA-positive patients, independent of biologic use, whereas in ACPA-negative patients an inverse association between BMI and cardiovascular events was observed only among biologic users [53].

Other studies also show that obesity’s impact varies substantially across therapeutic classes. This variation potentially reflects how different drug mechanisms are vulnerable to obesity-associated perturbations. Patients with obesity demonstrate markedly reduced response rates to TNF inhibitors, with only 35% achieving low disease activity compared to 72% of normal-weight patients after 6 months of anti-TNF therapy [54]. One hypothesis is that adipose tissue continuously supplies TNF at levels that overwhelm inhibitor capacity, with, for example, secondary upregulation of IL-6 and IL-1β signaling [55].Conversely, some JAK inhibitors (which target intracellular signaling pathways rather than extracellular cytokines) maintain efficacy across BMI categories, suggesting that inhibiting shared intracellular pathways may circumvent the redundancy created by obesity’s pleiotropic inflammatory effects [56]. However, this finding needs replication across different JAK inhibitors and patient populations before drawing firm conclusions.

### 5.2. Psoriatic Arthritis: Mechanistic Complexity and Variable Treatment Responses

In PsA, obesity’s interference with treatment efficacy reflects the disease’s unique immunopathology, wherein the IL-23/IL-17A axis has a more important role than in RA. In PsA, a cross-sectional analysis of 557 PsA patients revealed that obesity significantly reduced the likelihood of achieving remission across multiple validated criteria [36]. The ReFlap study revealed that PsA patients with a BMI ≥ 30 kg/m^2^ were less likely to achieve minimal disease activity [57]. Registry studies show also that PsA patients with obesity require more frequent treatment switching, with TNF inhibitor persistence rates declining proportionally with increasing BMI categories [58]. Little evidence is available for other therapy modalities in PsA, namely Il-17A and IL-23 inhibitors. Pantano et al. show in a small study of 100 PsA patients that secukinumab (IL-17A inhibitor) response does not differ based on BMI. Patients with PsA and obesity did show higher levels of IL-17A in peripheral blood [59]. The mechanistic basis for obesity’s effects in PsA likely involves obesity’s amplification key cytokines in PsA pathogenesis, including TNF, IL-6, IL-17A and IL-23. Contrarily to RA, JAK inhibitors show variable results in patients with PsA, with pooled analyses suggesting reduced efficacy, particularly in patients with BMI ≥ 35 kg/m^2^ [60]. This discordance between RA and PsA may reflect disease-specific differences: JAK/STAT signaling plays distinct roles in IL-23/IL-17A-driven inflammation (dominant in PsA) versus TNF/IL-6-driven pathology (more prominent in RA), and obesity may differentially impact these signaling networks. Additionally, JAK inhibitors’ effects on cellular glucose metabolism [61], which could theoretically benefit the metabolic dysfunction in obesity, may be insufficient to overcome the profound IL-17A-driven inflammation in PsA patients with obesity.

### 5.3. Gout: Metabolic Interference with Urate-Lowering Therapy

In gout, obesity compromises therapeutic efficacy through predominantly metabolic rather than immunologic mechanisms, creating a treatment-resistant phenotype distinct from but analogous to other inflammatory arthritides. A prospective cohort study of 633 male primary gout patients revealed that obesity substantially impairs urate-lowering therapy (ULT) effectiveness with febuxostat: while 63.8% of normal-weight patients achieved target serum urate levels (<6.0 mg/dL) after 12 weeks, only 54.2% of overweight and 38.9% of patients with obesity reached this goal [62]. Obesity’s interference with ULT efficacy seems multifactorial. Increased body weight alters drug pharmacokinetics through expanded volume of distribution and enhanced renal clearance, and reduces drug concentrations at target sites. Additionally, patients with obesity demonstrated higher baseline serum urate levels (9.40 mg/dL) and greater prevalence of metabolic comorbidities including hypertension (71.6%), hypertriglyceridemia (62.5%), and fatty liver disease (56.7%) compared to individuals with BMI < 25 kg m^2^ [62]. Unlike RA and PsA where obesity primarily amplifies inflammatory signaling, in gout obesity creates a broader metabolic derangement that fundamentally alters urate homeostasis

## 6. Chronic Inflammatory State Caused by Obesity

### 6.1. Adipose Tissue as an Active Immunometabolic Organ

The accumulating evidence that obesity drives both inflammatory arthritis susceptibility and treatment resistance necessitates fundamental understanding of underlying mechanisms to design effective interventions. The normal adipose tissue represents one of the most dynamic and metabolically active organ systems in the human body, fundamentally disproving the outdated perception of fat as merely passive energy storage. Rather than a homogeneous lipid depot, adipose tissue comprises a complex network of specialized cell types working in concert to maintain metabolic homeostasis, regulate immune function, and coordinate systemic energy balance [63]. The main cellular component of the adipose tissue is the adipocyte. White adipocytes serve as the primary energy storage cells, characterized by large unilocular lipid droplets that expand and contract in response to nutritional status. These cells demonstrate remarkable plasticity, storing triglycerides during energy abundance and releasing free fatty acids during periods of energy deficit through carefully regulated lipolytic pathways [64]. The inflammatory potential of adipose tissue is mostly linked to white adipose tissue. White adipose tissue has different anatomical locations, which also differ functionally. Subcutaneous depots, located beneath the skin throughout the body, demonstrate greater metabolic flexibility and are generally associated with favorable metabolic profiles. In contrast, visceral adipose tissue, particularly the omental and mesenteric depots, exhibits heightened inflammatory propensity and stronger associations with metabolic dysfunction. Most murine and human studies focus on the visceral adipose tissue and its white adipocytes.

### 6.2. Adipose Tissue Immune Cell Populations in Obesity

Under physiological conditions, adipose tissue harbors both innate and adaptive immune cells. Adipocytes interact with these immune cells, for example, via cytokines. In patients with obesity, the number and size of adipocytes increase. The resulting metabolic stress in adipose tissue attracts more immune cells, including macrophages, monocytes, and T cells. This triggers chronic activation. The percentage of macrophages increases from <10% in lean tissue to 40–60% in adipose tissue [65]. Other pathogenic immune cells found in human adipose tissue of patients with obesity are T cells, such as CD4+ Th17 cells. Adipocyte–macrophage–Th17 cell interactions result in a chronic proinflammatory loop involving cytokines, such as IL-1β and IL-17A leading to this inflammatory cascade [66]. Murine studies also found that CD8 T cells in visceral adipose tissue, secreting interferon (IFN) alpha and gamma, expand under high-fat diets and worsen antigen-induced arthritis in mice [67]. Furthermore, visceral fat can functionally affect regulatory T cells [68]. Additional immune cells are also implicated, including neutrophils, early infiltrators that generate ROS and neutrophil extracellular traps [69], NK cells, which produce IFNγ and contribute to adipose inflammation [70], innate lymphoid cells, which secrete IL-17A and IL-22 [71], and B cells, which are increased in obesity and support local antibody production and cytokine secretion [72]. The inflammatory state is not limited to the local fat, but cytokines also enter the bloodstream and a range of pro-inflammatory cytokines, including IL1B, IL17A, IL-2, IFNalpha, gamma and TNF are increased in peripheral blood of individuals with obesity [73].

### 6.3. Two Pathways to Arthritis: Autoimmune Versus Local Metabolic–Inflammatory Mechanisms

Obesity appears to influence inflammatory arthritis through two partially overlapping but mechanistically distinct pathways, whose relative contributions vary by disease phenotype.

In autoantibody-positive forms of arthritis, obesity amplifies systemic autoimmunity through adipose-derived factors that potentiate adaptive immune responses. As outlined in Section 5.1, adipokines such as leptin, IL-6, and TNF enhance B-cell survival, promote autoantibody production, and facilitate immune-complex formation. In this context, obesity acts not merely as a comorbidity but as an active amplifier of autoimmune inflammation—consistent with the observation that patients with seropositive rheumatoid arthritis exhibit greater disease activity and reduced treatment responsiveness.

In contrast, autoantibody-negative or seronegative arthritides appear to be more strongly linked to the innate immune activation associated with visceral adiposity. Obese visceral adipose tissue can perpetuate inflammatory arthritis primarily via activation of innate immune cells and CD8^+^ T cells, rather than through classical adaptive autoimmune pathways [67].

However, since not all individuals with obesity develop arthritis, an additional trigger for arthritis development seems to be needed. The answer may not only lie in what adipose tissue produces, but also in what it obstructs.

In addition to the general and local pro-inflammatory state associated with obesity, other localized mechanisms may contribute to arthritis susceptibility. Emerging evidence suggests that obesity creates both mechanical and metabolic obstruction of lymphatic drainage—the system responsible for clearing inflammatory mediators, immune complexes, and cellular debris from tissues, including synovial spaces [16]. Mechanically, excess adipose tissue can compress lymphatic vessels, while metabolically, hyperinsulinemia and lipotoxicity impair the contractile function and endothelial barrier integrity, compromising drainage function.

## 7. Normal Architecture of the Lymphatic System

The lymphatic vasculature comprises a hierarchical network of progressively larger vessels, each structurally and functionally specialized for distinct physiological roles. This organization begins with blind-ended initial lymphatics (lymphatic capillaries) in peripheral tissues and culminates in major collecting ducts that return lymph to the venous circulation. Initial lymphatics consist of a single layer of loosely connected lymphatic endothelial cells (LECs) that lack both a continuous basement membrane and perivascular mural cells such as pericytes or smooth muscle cells [17,74]. This unique structural configuration optimizes these vessels for their primary function: the efficient uptake of interstitial fluid, macromolecules, pathogens, and immune cells from peripheral tissues.

**Figure 1:**
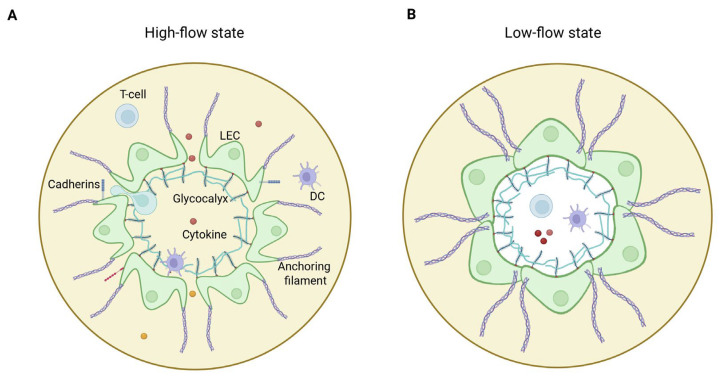
Architecture of initial lymphatics. (**A**) In high flow state, increased interstitial pressure opens flap valves, facilitating efficient fluid, (immune) cell, and protein entry and maintaining tissue fluid balance. The glycocalyx, a carbohydrate-rich layer, aids in mechanosensing and maintaining endothelial integrity. (**B**) In low flow state, reduced interstitial pressure closes flap valves, preventing retrograde flow and ensuring lymphatic vessel integrity. The glycocalyx contributes to regulating permeability and protecting endothelial cells. LEC = lymphatic endothelial cell; DC = dendritic cell.

The remarkable permeability of initial lymphatics results from specialized intercellular connections termed “button-like junctions” that form discontinuous, overlapping contact points between adjacent LECs (Figure 1) [75]. These junctions create “flap valves” that respond dynamically to changes in interstitial pressure. When tissue pressure increases, anchoring filaments connecting LECs to the extracellular matrix pull the overlapping cell borders apart, opening these primary valves to facilitate fluid entry (Figure 1A). Conversely, when intralymphatic pressure exceeds interstitial pressure, the flap valves close, preventing retrograde flow (Figure 1B). This pressure-sensitive inflow mechanism enables initial lymphatics to continuously clear excess interstitial fluid, maintaining tissue fluid balance while simultaneously sampling the tissue microenvironment for antigens, pathogens, and immune cells.

Recent discoveries have further revealed that LECs are covered by a glycocalyx [76], a carbohydrate-rich, multi-component layer previously well-characterized in blood vascular endothelium [76]. In blood vessels, the glycocalyx functions as a mechanosensor, responding to shear stress and flow dynamics [77]. Although its role in lymphatic vessels is not yet fully elucidated, it is hypothesized that the lymphatic glycocalyx similarly contributes to mechanosensing, complementing the pressure-sensitive valve system. Structurally, the glycocalyx is anchored to the endothelial surface via proteoglycans and glycoproteins, which not only stabilize the layer but also interact with the underlying vessel wall (Figure 1). Emerging evidence suggests that the thickness, molecular composition, and electrostatic charge of the glycocalyx may modulate endothelial permeability, adding an additional layer of regulation to lymphatic fluid entry and barrier function [76,77].

Initial lymphatics drain into pre-collecting vessels, which serve as transition zones where LECs begin to form tighter, continuous “zipper-like” junctions. These vessels subsequently merge with collecting lymphatics, which represent the mature, active transport segment of the lymphatic system and flow accelerates over these sections [74].

Interestingly, immune cells are only partially prone to active guidance to crawl from the initial lymphatics to the pre-collecting and collecting lymph vessels under physiological circumstances. The LECs in initial lymphatic play an active role in this process. LECs actively secrete chemokines, such as CCL21, and scavenging chemokine receptors (ACKRs), to guide migrating T cells from the initial lymphatics to the collector vessels [78]. However, T cells and also other immune cells, such as dendritic cells (DCs), can also linger in the initial lymphatics and crawl in and out, suggesting active interaction between T cells, dendritic cells and LECs [79]. Once immune cells enter the collecting vessels, flow picks up, under influence of specialized lymphatic smooth muscle cells (LSMCs) that provide contractile function essential for lymph transport, and intraluminal valves, preventing backflow. These intraluminal valves consist of two leaflets formed by specialized LECs expressing high levels of transcription factors including FOXC2, PROX1, and GATA2 [17,18,80]. Valve development and maintenance depend on mechanical forces, particularly oscillatory shear stress generated by lymph flow. All collecting vessels ultimately drain into lymph nodes. The lymphatic system incorporates over 200 lymph nodes in humans, strategically positioned along lymphatic routes to serve dual functions as filtration barriers and immune surveillance centers [17,78].

## 8. Obesity Impairs Lymphatic Function

### 8.1. Experimental Models of Lymphatic Dysfunction in Obesity

Murine models of diet-induced obesity have provided mechanistic insights into how obesity disrupts normal lymphatic function. In high-fat diet–fed mice, lymphatic collector vessels exhibit reduced contraction frequency, impaired valve function, increased permeability, leading to inefficient lymph transport [81,82]. Microlymphangiography demonstrated that diminished lymphatic transport changes lymph node architecture and cell composition and impairs dendritic cell migration [83].

Amongst other mechanistic explanations, perilymphatic accumulation of inflammatory macrophages and T cells, along with inducible nitric oxide synthase (iNOS) activity, drives LEC injury and progressive vessel dysfunction [84,85]. Notably, obesity-resistant strains maintained on the high-fat diet are protected from lymphatic impairment, underscoring that excess adiposity separate from diet composition may drive this pathology [86]. Encouragingly, weight loss restores contractile activity and reduces perilymphatic inflammation, suggesting the reversibility of these defects [85].

### 8.2. Disruption of Endothelial Cell Signaling at the Glycocalyx–Cell–Matrix Interface

Obesity-induced inflammation can compromise LEC function also through other mechanisms. Cytokines produced by adipose tissue, predominantly IL-4, IL-13, interferon-γ, and TGF-β, have been shown in vitro and in vivo to impair LEC function, migration, tubule formation, and active pumping [87,88,89]. Importantly, evidence from vascular endothelium studies supports the notion that obesity alters glycocalyx architecture. Fancher et al. demonstrated that visceral obesity in high-fat diet-fed mice significantly reduced glycocalyx thickness in mesenteric arteries, which was associated with impaired vascular flow. Complementary human data from fat biopsies obtained during bariatric surgery revealed similar glycocalyx alterations in patients with obesity, suggesting that these structural changes are conserved across species and vascular beds [90]. Although these findings pertain to blood vessels, the structural and functional parallels with LECs imply that similar glycocalyx degradation may occur in lymphatic vessels, contributing to increased permeability and impaired immune cell trafficking. Together, these molecular insults disrupt the glycocalyx–cell–extracellular matrix interface, undermining lymphatic barrier function and compromising the system’s ability to maintain tissue homeostasis.

### 8.3. Obesity-Associated Lymphatic Injury

The interaction between immune cells, circulating adipokines, cytokines and free-fatty acids and LECs is central to obesity-induced lymphatic dysfunction. iNOS, produced by macrophages and T cells surrounding lymphatic vessels in adipose tissue, damages LECs and increases permeability. Rehal et al. further tested the effect of iNOS on LEC function specifically in murine mesenteric lymphatic vessels, finding an increased number of iNOS+ immune cells around lymphatic vessels in high-fat diet-fed mice and demonstrating that LECs are highly sensitive to the damaging effects of iNOS production [82].

Adipokines such as leptin can inhibit LEC proliferation and tube formation in vitro [91]. Furthermore, chronic exposure to hypercholesterolemia results in structural lymphatic abnormalities and impaired migration of dendritic cells towards the draining lymph node as priming stage of the multistep immune response [92]. Finally, free fatty acids, abundantly present in adipose tissue, are toxic to LECs even at low concentrations, resulting in reduced proliferation, downregulation of key LEC genes, and direct apoptosis [86]. Thus, obesity creates a multi-pronged assault on lymphatic function through inflammatory, metabolic, and direct cytotoxic mechanisms that collectively compromise the lymphatic system’s capacity to maintain tissue homeostasis.

### 8.4. Obesity and Lymphatic Function in Human Studies

Human studies consistently support the concept that obesity impairs lymphatic function. In a seminal tracer study, participants with obesity exhibited reduced clearance of radiolabeled macromolecules from subcutaneous adipose tissue compared with lean controls, indicating compromised lymphatic drainage capacity [93]. Case reports have described ‘obesity-induced lymphedema,’ in which extreme obesity resulted in multi-limb lymphatic failure, as documented by lymphoscintigraphy [94]. Larger clinical series confirmed that individuals with severe obesity develop lymphoscintigraphic abnormalities, including dermal backflow and absent nodal tracer uptake, most often affecting the lower extremities [94]. Importantly, retrospective analyses demonstrate that the likelihood of abnormal lymphatic function rises sharply with increasing body mass index, with near-universal impairment observed in patients with severe obesity [95]. Collectively, these findings suggest that obesity not only predisposes to lymphedema but also induces systemic alterations in lymphatic transport, providing a mechanistic link between adiposity, impaired immune cell trafficking, and chronic tissue inflammation.

### 8.5. Specific Dysfunction in Mesenteric Lymphatic Vessels

Beyond its systemic effects on the lymphatic vasculature, obesity appears to create specific dysfunction in the mesenteric lymphatic vessels, based on findings from murine studies. These vessels drain both the visceral adipose tissue and the small intestine. High-fat diet-fed mice exhibit impaired active pumping of the mesenteric lymphatic vessels, resulting in lymph stasis [20]. Notably, the murine mesenteric lymph nodes, in contrast to subcutaneous lymph nodes, expand and show increased immune cells with shifted populations, particularly a marked decrease in regulatory (CD4+Foxp3+) T cells, indicating a shift towards a pro-inflammatory milieu [20,21]. Lymph from high-fat diet-fed mice stimulates mesenteric lymphatic vessel growth but leads to ineffective, highly branched lymphatic vessels that leak lipid-rich lymph into the visceral adipose tissue [96,97]. Moreover, damaged mesenteric lymphatic vessels may institute a vicious cycle, since mesenteric lymphatic dysfunction may independently contribute to adiposity. Earlier studies demonstrated that Prox1± heterozygous mice, deficient in the key transcription factor for LECs, develop severe lymphatic defects, specifically in the mesentery, resulting in adipogenesis and adult-onset obesity [98]. These murine findings underscore potential disruption of mesenteric lymphatics resulting in worsening visceral adipose tissue inflammation and contributing to the chronic pro-inflammatory milieu. However, detailed studies on human obese mesenteric lymphatics examining lymph node size, number, or immune cell composition are currently lacking. Collectively, these findings underscore the multifaceted impact of obesity on lymphatic function, highlighting the critical need for targeted therapeutic strategies to mitigate lymphatic dysfunction and its associated complications in patients with obesity.

## 9. Obesity-Induced Local Lymphatic Injury as a Contributing Factor to Chronic Joint Inflammation

Another critical link between obesity, lymphatic dysfunction and inflammatory arthritis lies in the joint’s dependence on efficient lymphatic clearance for resolution of inflammatory episodes. Lymphatics in healthy synovial tissue rapidly drain inflammatory mediators, immune complexes, and activated immune cells from the joint space to regional lymph nodes, facilitating both the termination of inflammatory responses and the restoration of tissue homeostasis [13,99]. This concept is supported by experimental evidence in murine models of chronic inflammatory arthritis using TNF transgenic mice, inhibiting lymphatic drainage significantly exacerbated arthritis severity [100,101]. Anti TNF therapy ameliorates arthritis by restoring lymphatic transport and relieving physical lymphatic obstruction by immune cells [101]. A small comparative human study of 8 RA patients and 13 healthy controls using indocyanine green (ICG) dye and near-infrared (NIR) lymphatic imaging revealed reduced lymphatic drainage and fewer lymphatic vessels in the hands of active RA patients [102]. Aldrich et al. also demonstrated dermal backflow in lymphatics in an inflammatory arthritis patient [103]. Additional clinical studies on lymphatic imaging are needed to strengthen the hypothesis of impaired lymphatic drainage in joints affected by arthritis. Given that obesity systemically impairs lymphatic contractility, increases vessel permeability, and compromises valve function [81,82], the consequent defects would also negatively influence lymphatic drainage of the synovial cavity, creating conditions that favor persistent joint inflammation.

**Figure 2:**
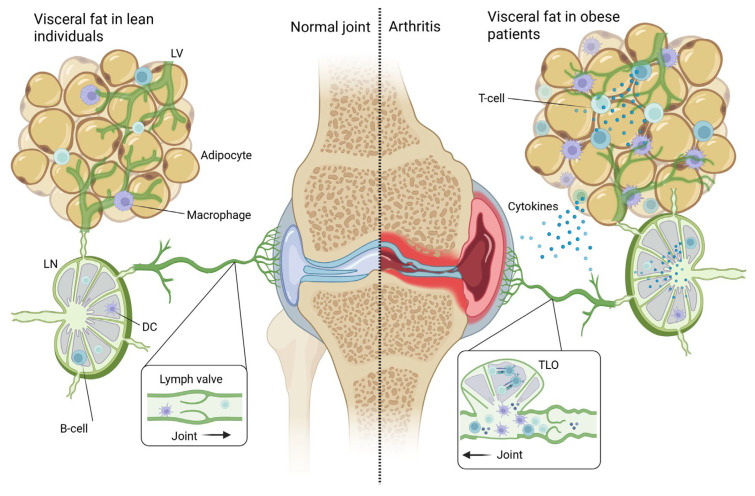
Schematic comparison of normal versus obesity-associated inflammatory arthritis. **Left panel:** Healthy joint with functional lymphatic drainage. Normal synovium maintains tissue homeostasis through efficient lymphatic clearance of inflammatory mediators and immune cells via patent collecting vessels with functional intraluminal valves. Normal visceral adipose tissue exhibits minimal immune cell infiltration and low cytokine production. **Right panel:** Inflammatory arthritis joint with impaired lymphatic function as seen in obesity. Inflamed synovium accumulates inflammatory mediators due to compromised lymphatic drainage. The collecting lymphatic vessel shows structural deterioration with valve dysfunction and physical obstruction. Tertiary lymphoid organ (TLO) formation near the dysfunctional valve creates a pathological feedback loop, further obstructing lymphatic flow and perpetuating chronic inflammation. In obesity visceral adipose tissue demonstrates marked immune cell infiltration (macrophages, T cells) and elevated pro-inflammatory cytokine production (TNF, IL-6, IL-1β, IL-17A), contributing to both systemic inflammation and local lymphatic impairment. This model integrates obesity-induced adipose dysfunction, lymphatic impairment, and TLO formation as interconnected mechanisms promoting inflammatory arthritis chronicity. LN = lymph node; LV = lymphatic vessel; DC = dendritic cell; TLO = tertiary lymphoid organ.

Beyond mechanical impairment, obesity-induced lymphatic dysfunction creates the requisite micro environmental conditions that may promote tertiary lymphoid organ (TLO) formation, including chronic antigen presentation, sustained inflammatory signaling, and compromised immune cell trafficking. TLO formation in synovial tissue is well-documented in inflammatory arthritis, particularly in rheumatoid arthritis, where Thurlings et al. found TLOs present in one-third of patients, correlating with elevated CRP and leukocyte levels despite no significant increase in clinical disease severity [104]. The link between lymphatic dysfunction and TLO formation is most clearly demonstrated in Crohn’s disease, where TLOs originate specifically at lymphatic valves and directly obstruct mesenteric lymph flow. In their ileitis model, TNF exposure downregulated lymphatic valve maintenance genes, causing valve dysfunction and creating permissive sites for TLO formation that subsequently restricted lymphatic drainage and immune cell migration. This establishes a pathological feedback loop: initial lymphatic impairment enables TLO establishment, which then further compromises lymphatic function and perpetuates chronic inflammation **(**Figure 2) [105,106,107]. Critically, while early TNF neutralization could restore lymphatic transport, robustly developed TLOs resisted regression even after TNF blockade, indicating that once established, these structures become self-sustaining drivers of disease chronicity. We propose that this lymphatic valve-initiated TLO formation observed in Crohn’s disease may represent a convergent pathway relevant to obesity-associated inflammatory arthritis, potentially linking adiposity, lymphatic dysfunction, and disease chronicity. However, several critical knowledge gaps limit this interpretation. While TLO formation has been extensively characterized in rheumatoid arthritis synovium, comprehensive studies examining TLO prevalence, formation mechanisms, and functional significance in other inflammatory arthropathies—including psoriatic arthritis and gout—remain limited. More importantly, direct evidence demonstrating that obesity-induced lymphatic dysfunction specifically promotes TLO formation in human arthritic joints is currently lacking. Whether the lymphatic–TLO axis observed in Crohn’s disease translates to the pathophysiology of inflammatory arthritis, and whether this mechanism specifically mediates obesity’s effects on disease chronicity, requires dedicated investigation.

## 10. Incretin Analogs as Dual-Pathway Disease-Modifying Therapeutic Options

Incretin analogs, such as GLP-1 agonists, are increasingly used to treat diabetes and obesity [108]. Limited studies are performed in inflammatory arthritis, but the first results indicate a positive effect of GLP-1 agonist on reducing systemic inflammatory cytokines and improving metabolic parameters as reviewed recently [109]. The effect seems to be predominantly linked to weight loss, but not all underlying mechanisms are clear yet. Mechanistically, GLP-1 agonists demonstrate evident anti-TNF activity through NF-κB pathway inhibition, with exendin-4 significantly reducing TNF levels induced by multiple Toll-like receptor agonists and liraglutide decreasing synovial inflammation and pro-inflammatory cytokine production in a murine osteoarthritis model [110,111]. The anti-hyperinsulinemic effects are well-established, with substantial weight loss (24% body weight in lymphedema patients) and improved insulin sensitivity through PKA/CREB signaling activation [108]. More speculatively, emerging evidence suggests direct lymphatic benefits independent of weight loss, with GLP-1 agonists reducing lymphedema risk following axillary lymph node dissection and demonstrating return of lymphatic flow on imaging studies [112]. Given the current evidence—limited clinical data in inflammatory arthritis, predominantly preclinical mechanistic studies, and uncertainty about direct versus indirect effects—it is premature to characterize GLP-1 agonists as disease-modifying therapy in inflammatory arthritis. Nevertheless, the convergence of anti-inflammatory, metabolic, and potentially lymphatic effects observed across disease models provides scientific rationale for rigorous investigation of incretin analogs in patients with obesity-associated inflammatory arthritis. Well-designed clinical trials with arthritis-specific outcome measures, mechanistic endpoints, and weight loss-matched control groups are essential to determine whether these agents offer disease-modifying potential beyond their established metabolic benefits. Importantly, these pharmacological approaches must complement rather than replace foundational therapeutic elements. Clinicians should prioritize comprehensive management strategies that couple immunosuppressive therapies with evidence-based weight management interventions, including physician-supervised dietary modification and individually tailored exercise regimens, acknowledging that successful treatment of obesity-associated inflammatory arthritis requires simultaneous targeting of inflammatory and metabolic axes.

## 11. Concluding Remarks

The intersection of obesity and inflammatory arthritis represents a growing clinical and scientific challenge. The global obesity epidemic substantially overlaps with the rising prevalence of inflammatory arthritis, yet current therapeutic approaches largely treat obesity and arthritis as independent conditions. This perspective synthesizes emerging evidence that obesity drives inflammatory arthritis through multiple, overlapping pathways operating across systemic and local anatomical levels (Figure 2). Systematically, obese visceral adipose tissue generates chronic low-grade inflammation through secretion of pro-inflammatory cytokines (TNF, IL-6, IL-1β) and adipokines, creating a permissive immunological environment that both may predispose to disease initiation and can amplify established arthritis. This systemic inflammatory state potentiates autoimmune responses in ACPA-positive RA through adipokine-mediated enhancement of B cell activation and autoantibody production. Simultaneously, at the local visceral adipose tissue level, dysfunctional visceral adipose tissue activates innate immune cells and, for example, CD8+ T cells by continuous exposure of metabolic stress signals and inflammatory mediators.

Simultaneously, at the local tissue level, visceral obese adipose tissue directly damages local lymphatic vessels through multiple mechanisms: glycocalyx degradation, impaired lymphatic smooth muscle contractility due to hyperinsulinemia and lipotoxicity, and mechanical compression from expanding adipose depots. This initiates a devastating regional feedback loop wherein impaired lymphatic drainage from visceral fat reduces immune cell trafficking to lymph nodes, trapping activated immune cells and inflammatory mediators within the adipose tissue itself, thereby intensifying and perpetuating local chronic inflammation. The resulting chronic inflammatory environment further compromises lymphatic vessel structure and function, deepening the vicious cycle of adipose tissue inflammation and lymphatic insufficiency.

Synovial inflammation generates high concentrations of TNF, IL-1β, and matrix metalloproteinases that degrade lymphatic endothelial glycocalyx, disrupt button junction integrity, and impair valve function in joint-draining lymphatics. This local lymphatic failure creates a second vicious cycle: inadequate clearance of inflammatory mediators, immune complexes, and activated immune cells from the synovial space sustains chronic joint inflammation, which further damages lymphatic structures. The accumulation of immune cells and chronic antigen presentation in this context of failed lymphatic clearance creates permissive microenvironmental conditions for tertiary lymphoid organ (TLO) formation—often at or near dysfunctional lymphatic valves, as demonstrated in Crohn’s disease models. These ectopic lymphoid structures physically obstruct remaining functional lymphatics while serving as autonomous sites of autoantibody production and immune cell activation, completing a self-perpetuating inflammatory circuit that resists conventional immunosuppressive therapies. Thus, obesity and inflammatory arthritis interact through nested, reinforcing feedback loops across multiple anatomical scales, explaining both increased disease susceptibility and treatment-resistant phenotypes in patients with obesity.

Incretin analogs emerge as uniquely positioned therapeutic agents that simultaneously address multiple mechanisms in obesity-induced inflammatory arthritis: reducing obesity-driven inflammation, correcting hyperinsulinemia, restoring lymphatic function, and potentially preventing TLO establishment. Their dual metabolic and anti-inflammatory properties suggest disease-modifying potential that extends beyond symptom management to address fundamental pathophysiological drivers. Combining current immunosuppressive agents with incretin analogs could provide transformative therapeutic benefits for this challenging patient population. However, current evidence—predominantly preclinical—necessitates cautious interpretation. These agents should be conceptualized as one component within comprehensive strategies integrating immunomodulatory therapies, lifestyle interventions, and metabolic pharmacotherapy rather than standalone disease-modifying treatments.

Research priorities include strengthening fundamental investigations of lymphatic dysfunction in obesity and its underlying mechanisms, as well as demonstrating lymphatic impairment in visceral adipose tissue and synovium in inflammatory arthritis. Defining the optimal timing for intervention to preserve lymphatic architecture and maybe preventing TLO formation will be critical for designing effective combination therapies. In summary, recognizing lymphatic dysfunction as a critical mechanism in obesity-related arthritis offers hope for transforming outcomes in a challenging population.

## Data Availability

All data are contained within the article.

## Abbreviations

The following abbreviations are used in this manuscript:

ACKRs	Atypical chemokine receptors
ACPA	Anti-citrullinated protein antibodies
ALND	Axillary lymph node dissection
BMI	Body mass index
BRI	Body roundness index
CCL21	C-C motif chemokine ligand 21
CDAI	Clinical Disease Activity Index
CRP	C-reactive protein
CSA	Clinically suspect arthralgia
DAPSA	Disease Activity in Psoriatic Arthritis
DAS28	Disease Activity Score in 28 joints
DCs	Dendritic cells
ESR	Erythrocyte sedimentation rate
FOXC2	Forkhead box protein C2
GATA2	GATA binding protein 2
GLP-1	Glucagon-like peptide-1
HAQ	Health Assessment Questionnaire
IFN	Interferon
IL-1β	Interleukin-1 beta
IL-2	Interleukin-2
IL-4	Interleukin-4
IL-6	Interleukin-6
IL-13	Interleukin-13
IL-17	Interleukin-17
IL-17A	Interleukin-17A
iNOS	Inducible nitric oxide synthase
JAK	Janus kinase
LECs	Lymphatic endothelial cells
LSMCs	Lymphatic smooth muscle cells
MSU	Monosodium urate
NF-κB	Nuclear factor kappa B
NHANES	National Health and Nutrition Examination Survey
OR	Odds ratio
PKA/CREB	Protein kinase A/cAMP response element-binding protein
PROX1	Prospero homeobox protein 1
PsA	Psoriatic arthritis
RA	Rheumatoid arthritis
RF	Rheumatoid factor
RR	Relative risk
SDAI	Simplified Disease Activity Index
TGF-β	Transforming growth factor beta
Th17	T helper 17 cells
TLO	Tertiary lymphoid organ
TLOs	Tertiary lymphoid organs
TLR	Toll-like receptor
TNF	Tumor necrosis factor
TNF-α	Tumor necrosis factor alpha
ULT	Urate-lowering therapy
VFO	Visceral fat obesity
WWI	Weight-adjusted waist index
